# Dining Bowl Modeling and Optimization for Single-Image-Based Dietary Assessment

**DOI:** 10.3390/s24186058

**Published:** 2024-09-19

**Authors:** Boyang Li, Mingui Sun, Zhi-Hong Mao, Wenyan Jia

**Affiliations:** 1 Department of Electrical & Computer Engineering, University of Pittsburgh, Pittsburgh, PA 15260, USA; bol33@pitt.edu (B.L.); drsun@pitt.edu (M.S.); zhm4@pitt.edu (Z.-H.M.); 2 Department of Bioengineering, University of Pittsburgh, Pittsburgh, PA 15260, USA; 3 Department of Neurological Surgery, University of Pittsburgh, Pittsburgh, PA 15260, USA

**Keywords:** image-based dietary assessment, bowl modeling, 3D reconstruction, optimization

## Abstract

In dietary assessment using a single-view food image, an object of known size, such as a checkerboard, is often placed manually in the camera’s view as a scale reference to estimate food volume. This traditional scale reference is inconvenient to use because of the manual placement requirement. Consequently, utensils, such as plates and bowls, have been suggested as alternative references. Although these references do not need a manual placement procedure, there is a unique challenge when a dining bowl is used as a reference. Unlike a dining plate, whose shallow shape does not usually block the view of the food, a dining bowl does obscure the food view, and its shape may not be fully observable from the single-view food image. As a result, significant errors may occur in food volume estimation due to the unknown shape of the bowl. To address this challenge, we present a novel method to premeasure both the size and shape of the empty bowl before it is used in a dietary assessment study. In our method, an image is taken with a labeled paper ruler adhered to the interior surface of the bowl, a mathematical model is developed to describe its shape and size, and then an optimization method is used to determine the bowl parameters based on the locations of observed ruler makers from the bowl image. Experimental studies were performed using both simulated and actual bowls to assess the reliability and accuracy of our bowl measurement method.

## 1. Introduction

There are growing concerns about high-calorie, low-nutrition diets and their long-term health complications, such as obesity, hypertension, diabetes, and heart disease. A crucial aspect in addressing these public health problems is to assess an individual’s nutrient and calorie intake quantitatively. The result of this assessment can provide data to aid in the prevention and treatment of various chronic diseases through personalized intervention and precision medicine [1]. Traditionally, dietary assessment is often conducted by self-reporting approaches (e.g., 24-h dietary recall [2], food diary [3], and food frequency questionnaire [4]), which are subject to bias and inaccuracy. With the increasing popularity of mobile devices, image-based dietary assessment methods using a smartphone or a wearable camera have been developed. These methods, which are mostly single-image-based, involve two important steps that are still being investigated: food recognition and portion size estimation. With the rapid development of deep learning techniques, food recognition studies have achieved significant progress [5,6,7,8,9,10,11]. However, accurately estimating food volume remains a significant challenge [10,11,12,13,14,15].

Besides unsolved problems with respect to the irregular shapes of various foods [16,17] (this topic is outside the scope of this paper), lacking an accurate reference to determine the size of the food in an image is currently problematic. This problem is analogous to the case where an accurate scale bar is lacking when the area of a region in a map is measured. There have been many forms of scale references studied. We divide commonly used references into two groups: traditional references and utensil references. Traditional references commonly involve the deliberate inclusion of a physical object of known size, such as a checkerboard [18,19,20,21], a rectangular object (a wallet, a card, etc.) [22,23], or a coin [24,25,26] alongside the food. This type of reference is inconvenient to use because of the required manual procedure of placing this reference in the camera’s view. In contrast, utensil references involve items like a spoon [27], chopsticks [28], and a food container—usually a bowl [29] or a plate [30]—which provide essential reference information for estimating food volume without the need for a special manual procedure. For a shallow circular plate, the plate diameter is usually used as the scale reference, and the observed plate distortion (i.e., a round plate appears as an ellipse in the image when not viewed from directly above) is used to determine the camera’s orientation [30,31]. However, if the container is a bowl, the food may be obscured partially or completely by the edge of the bowl unless the image is taken from above the food. Thus, to estimate the nutrients/portion of food in a bowl, top-view images are used [16,32,33]. Although obscureness is avoided, food volume estimation cannot be accurate regardless of the estimation method used. This is because the bottom part of the food shape is determined by the shape of the bowl, but the bowl shape is unobservable from the image. To solve this problem, a spherical cap has been used to represent the bowl shape after the bowl’s height and diameter are estimated [28,34,35], which is not accurate.

Since the shape of food in a bowl is at least partially determined by the shape of the bowl, it is desirable to pre-measure the bowl to provide a more accurate reference to estimate the food volume. Jia et al. [29] proposed to pre-measure the size and shape of a bowl using an adhesive paper strip printed with ruler markers. When the bowl with the strip is photographed, the paper strip exhibits distortions, which are analyzed for bowl reconstruction. Although this method provides complete 3D information about the bowl, the photograph must be taken directly above the center of the bowl. In practice, it is difficult to keep the camera position exactly where it is required.

In this study, we focus on solving the size and shape measurement problem for the bowl so that it can be used as an accurate scale reference in the subsequent food volume estimation step (this step is not discussed in this paper but is described in [28,36]). We relax the requirement for the camera to be positioned directly above the center of the bowl. We incorporate a simple mathematical model to represent the shape/size of the bowl. Instead of computing the profile (curve) of the interior surface of the bowl point-by-point, we determine the parameters of our model using a coarse-to-fine numerical optimization procedure. Our method innovates the utensil reference technique for image-based dietary assessment.

This paper is organized as follows: Section 2 describes the mathematical model representing the bowl and outlines the algorithms for estimating the model parameters. Section 3 provides details of the experiments using both simulated and measured bowl data. Section 4 discusses several implementational issues of our method towards its practical application. Lastly, Section 5 draws conclusions.

## 2. Methods

In this section, we present a mathematical model that represents the shape of a circular bowl and an optimization algorithm for estimating bowl parameters.

### 2.1. Bowl Model

Although most real-life bowls are circular when viewed from the top, they have various shapes when viewed from the side, exhibiting characteristics ranging from a “thin bowl” to a “plump bowl” [37,38]. Consequently, we introduce a quantitative measure called “plumpness”, denoted by p, to systematically capture these characteristics:

(1) $$p=\frac{V_{bowl}}{V_{cylinder}}.$$

Equation (1) indicates that p is a ratio of the bowl’s volume $V_{bowl}$ to the volume of a cylinder, $V_{cylinder}$, with the same radius and height as the bowl. When the bowl is very “thin”, it approaches a spherical cone, $p\to \frac{1}{3}$ . On the other hand, when the bowl is very “plump”, it approaches a cylinder, p→1. Therefore, $\frac{1}{3}\;$and 1 define the lower and upper bounds of p , respectively.

**Figure 1:**
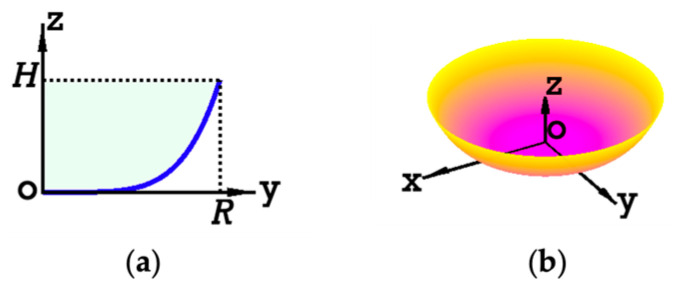
(**a**) Cross-section curve of the bowl model (blue), with the green-shaded region showing the area enclosed by the curve; (**b**) 3D bowl surface after rotating the curve in (**a**) by 360°, with the color of the surface varying according to the heights specified by Z.

Let *R* and H denote, respectively, the radius and height of the bowl. Let the central cross-section of the bowl in the y–z plane be z=f(y), where f(y) is a continuously differentiable and strictly monotonic function, as illustrated in Figure 1a. By rotating f(y) 360 degrees around the z-axis, a three-dimensional surface is formed (shown in Figure 1b), representing the interior surface of the bowl.

Given f(y), Equation (1) becomes

(2) $$p=\frac{\int_{0}^{H}\pi y^{2}dz}{\pi R^{2}H}=\frac{1}{R^{2}H}\int_{0}^{R}y^{2}\frac{df(y)}{dy}dy.$$

The last step in Equation (2) is obtained by changing the integration variable from z to y. Among various candidates for f(y), we aimed to choose a function that is simple yet reflects the shape of the bowl. After comparing several candidates, we selected a power function, given by

(3) $$z=f\left(y\right)={\left(\frac{y}{R}\right)}^{q}H,\;\mathrm{for}\;0\leq y\leq R,q>1$$

where q is a parameter that determines the bowl shape. We chose this particular function primarily for two reasons: (1) it is continuously differentiable and strictly monotonic; thus Equation (2) is well-defined, and (2) for q>1

(4) $$\frac{df(y)}{dy}=\frac{qy^{q-1}H}{R^{q}}\equiv 0\;at\;y=0.$$

Equation (4) ensures that the bowl meets an important requirement that it must be smooth and stable when placed on the “tabletop” (i.e., x–y plane) because the bowl has a nearly flat region at the “bottom” (Figure 1a).

Substituting Equation (3) into Equation (2), we have

(5) p=1R2H∫0Ry2df(y)dydy=1R2H∫0Ry2qyq−1HRqdy=qRq+2∫0Ryq+1dy=qRq+2yq+2q+2R0=qq+2.

**Figure 2:**
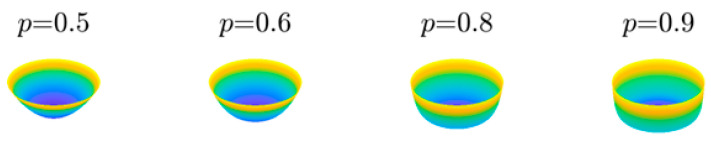
Bowl shapes with different values of plumpness (p) representing by surface plots, with the color of the surface varying according to the heights specified by Z.

Let us now determine the lower and upper bounds of p. When $q\to 1,\;p\to \frac{1}{3}$; when q→∞, p→1. Thus, these bounds (stated after Equation (1)) are correct. Figure 2 shows a few example bowls with different plumpness values.

### 2.2. Formulation of Parameter Estimation

**Figure 3:**
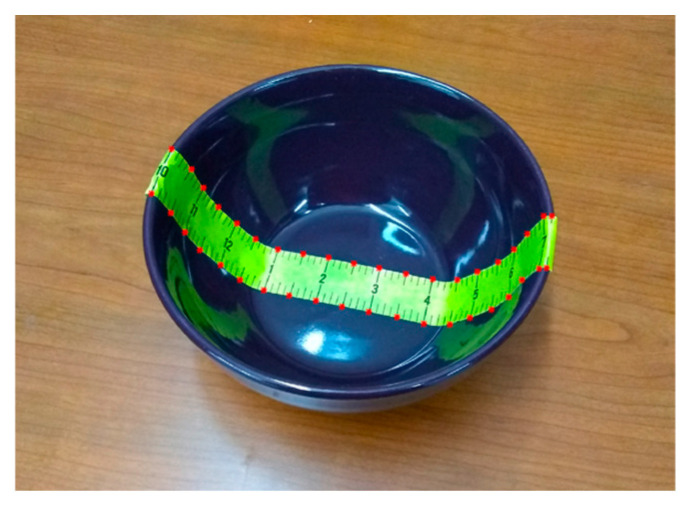
A bowl taped with an adhesive paper ruler. The red dots represent the selected landmark points.

The mathematical model of the bowl (Equation (3)) requires determining three parameters: rim radius *R*, height *H*, and shape parameter *q*. While *R* and *H* can be measured physically using a ruler, there is no simple way to determine *q*. To solve this problem, we attach an adhesive paper ruler centrally across the bottom of the bowl (Figure 3) and then take a picture using a smartphone. Upon close observation, it can be found that variations in both the width of the paper ruler and the spacing between the black division markers provide information about the bowl’s shape. Closer locations to the camera exhibit wider width and larger spacings, while the opposite is true for farther locations. Additionally, the width and spacing also vary with the camera’s view angle. As stated previously, we developed an image-processing algorithm to reconstruct the actual cross-section curve of the bowl without using a mathematical model [29]. However, due to the limitations of that algorithm, the picture must be taken directly above the bowl. Unfortunately, this form of picture-taking is challenging in practice because finding the correct top view by manually positioning the smartphone is difficult. In the following pages, we present an optimization method to estimate the bowl shape without the top-view requirement, simplifying the practical procedure for bowl measurement.

We first created two groups of landmark points from the observed paper ruler in the 2D input image. One group is located along the upper edge of the ruler, and the other is positioned along the bottom. For example, in the case shown in Figure 3, except for the points at both ends, we chose landmark points (e.g., all 0.5-inch markers). Let $c_{i}$ denote the coordinates (in terms of pixels) of the *i*th landmark point obtained from the 2D input image:

(6) $$c_{i}=\left[\begin{array}{c} m_{i}\\ n_{i} \end{array}\right].$$

Here, i=1, 2,⋯,N, where N is the total number of landmark points. Let $p_{i}={\left[x_{i},y_{i},z_{i}\right]}^{T}\;$denote the coordinates of the 3D point on the inner surface of the bowl that corresponds to $c_{i}$, with $z_{i}={\left(\frac{\sqrt{{x_{i}}^{2}+{y_{i}}^{2}}}{R}\right)}^{q}H$ (according to Equation (3) after rotation around the *z*-axis). Now, we apply the standard camera coordinate transformation to project $p_{i}$ onto the 2D image $p_{i}$ [39,40,41]. The reprojection error, which is the discrepancy between the landmark point $c_{i}$ and the projection of the simulated 3D point $p_{i}$, depends on the accuracy of the bowl’s model and the camera’s extrinsic parameters. Minimizing this discrepancy enables us to find the best 3D points that match the landmark points observed from the image [42], thereby allowing us to determine the shape of the bowl. Specifically, in the first step, a 3D point $p_{i}$ in the physical world is transformed into a 3D point $p_{i}^{\prime }={\left[x_{i}^{\prime },\;y_{i}^{\prime },z_{i}^{\prime }\right]}^{T}$ in the camera coordinate system by

(7) $$p_{i}^{\prime }=Rp_{i}+t$$

where R is a rotation matrix and tis a three-dimensional translation vector. In the next step, the 3D point $p_{i}^{\prime }\;$is projected onto the 2D image as ${\tilde{c}}_{i}={\left[{\tilde{m}}_{i},{\tilde{n}}_{i}\right]}^{T}$ using the ideal pinhole camera model [40]:

(8) $${\tilde{c}}_{i}=\left[\begin{array}{c} {\tilde{m}}_{i}\\ {\tilde{n}}_{i} \end{array}\right]=\left[\begin{array}{c} F\frac{x_{i}^{\prime }}{z_{i}^{\prime }}{+c}_{x}\\ F\frac{y_{i}^{\prime }}{z_{i}^{\prime }}{+c}_{y} \end{array}\right]$$

where F is the focal length of the camera in pixels and $c_{x}\;and\;c_{y}$ represent the *x*- and *y*- coordinate, respectively, of the optical center (also known as the principal point) in the image. Note that F, $c_{x},and\;c_{y}$ are intrinsic camera parameters, usually obtained from a camera calibration process [43].

At this point, we have two sets of 2D points, denoted as $c_{i}$ and ${\tilde{c}}_{i}$, corresponding to the measured landmark points and the projected 2D points, respectively.We aimed to solve the following optimization problem:

(9) $$\underset{S}{\min}J=\sum_{i=1}^{N}{\left\|{\tilde{c}}_{i}-c_{i}\right\|}^{2}$$

where S={R,H,q,R,t}is the set of parameters to be optimized and $\left\|\cdot \right\|$ denotes the Euclidean norm.

### 2.3. Numerical Optimization

**Figure 4:**
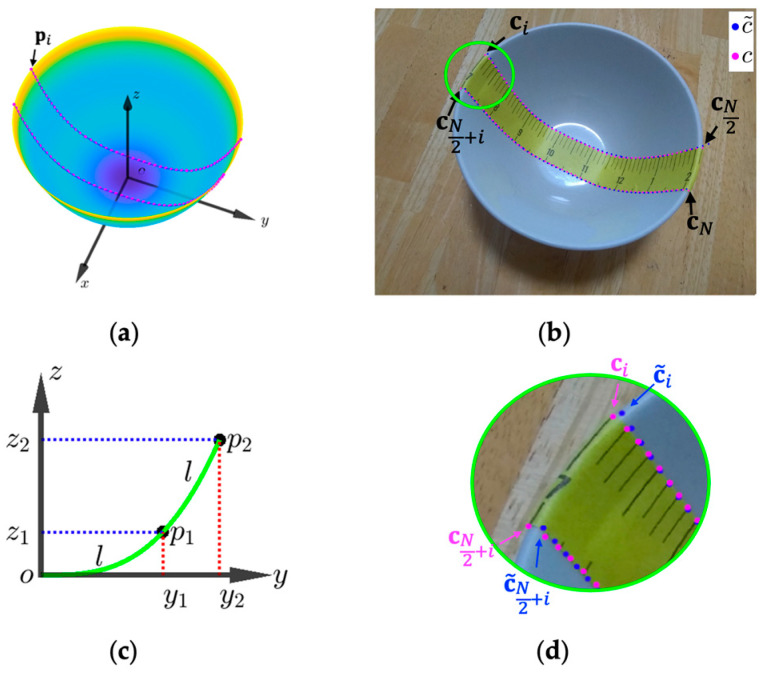
(**a**) Three-dimensional bowl surface with 3D landmarks $p_{i}$ highlighted in magenta; (**b**) estimated 2D projected landmarks ${\tilde{c}}_{i}$ in blue vs. real landmarks $c_{i}$ in magenta; (**c**) cross-sectional view of the tape’s edge represented by a green curve in the 3D space, where l denotes equal-length distances between markers on the adhesive paper ruler in the 3D space. Points $p_{1\;}$and $p_{2}$ are 3D points with coordinates ($y_{1},$ $z_{1}$) and ($y_{2}$, $z_{2}$); (**d**) enlargement of the green circle from Figure 4b, illustrating ${\tilde{c}}_{i}$ in blue and $c_{i}$ in magenta.

To estimate the bowl’s parameters—H,q, and R—through one-step optimization, it is imperative to express the 3D landmarks, $p_{i},\;i=1,\;2,\cdots ,N$ (shown in Figure 4a) and then use the standard camera coordinate transformation to project $p_{i}$ to the measured 2D points, $c_{i}.$ However, $p_{i}$ is a function of H,q, and R, which are all target variables for optimization, and we cannot express this function in a closed form (we will revisit this problem in Section 3.1). As a result, we cannot estimate the bowl’s parameters through a simple one-step optimization process.

Thus, we propose a coarse-to-fine strategy to solve the optimization problem by estimating the camera pose, which includes its rotation matrix R (represented by Euler angles ϕ, θ, and ψ) and translation vector $t={\left[X,Y,Z\right]}^{T}$ (representing the camera’s movements along the *x*-, *y*- and *z*-axes, respectively). The specific steps of our proposed coarse-to-fine optimization algorithm are as follows:

1. Define a coarse grid for H,q, and R, where each point on the grid corresponds to a bowl model.
2. For each bowl model, obtain the optimal camera pose using the Levenberg–Marquardt (LM) algorithm.
3. Find m candidate bowls based on the selection criteria defined in Equations (10)–(12).
4. For each candidate bowl, use random search to explore the neighborhood of H,q, and R, and use the LM algorithm to optimize the camera pose.
5. If the smallest error is less than a preset threshold (determined experimentally), then stop; otherwise, go back to step 3.

The selection criteria used in this work are defined as the sum of two errors.

The first error, denoted as the relative tape length error $e_{l}$, is defined as

(10) $$e_{l}=\left|\frac{L_{est}-L_{gt}}{L_{gt}}\right|$$

where $L_{est}$ and $L_{gt}$ denote the estimated and ground truth tape length, respectively.

The second error, denoted as the mean normalized reprojection error $e_{c}$, measures the normalized disparity between $c_{i}$(obtained from the 2D image) and the estimated landmarks ${\tilde{c}}_{i}$. It is defined as

(11) $$e_{c}=\frac{2}{N}\sum_{i=1}^{\frac{N}{2}}\frac{\left\|{{\tilde{c}}_{i}-c}_{i}\right\|+\left\|{{\tilde{c}}_{\frac{N}{2}+i}-c}_{\frac{N}{2}+i}\right\|}{\left\|c_{i}-c_{\frac{N}{2}+i}\right\|}.$$

Here, N is the total number of detected landmarks along the two sides of the paper ruler, with $\frac{N}{2}$ landmarks on each side (as shown in Figure 4b,d). The expression within the summation computes the error for each point as the difference between the estimated and observed positions, relative to the width of the tape. Since the relative errors of these $\frac{N}{2}$ pairs of points are summed, the total is divided by $\frac{N}{2}$ to obtain the average relative error. Then, the sum of these two errors serves as the selection criteria in our algorithm, defined as

(12) $$e_{n}=e_{c}+e_{l}.$$

### 2.4. Volumetric Error Analysis

As stated previously, the goal of bowl measurement is to estimate food volume. Therefore, after the three bowl parameters (*q*, *H*, and *R*) are estimated, it is important to understand the statistical properties of the bowl volumetric error in relation to the errors of these three estimates.

From basic estimation theory [44], we have

(13) $$\Delta V=V-V_{0}\;\;\frac{\partial V\left(q_{0},H_{0},R_{0}\right)}{\partial H}\Delta H+\frac{\partial V\left(q_{0},H_{0},R_{0}\right)}{\partial R}\Delta R+\frac{\partial V\left(q_{0},H_{0},R_{0}\right)}{\partial q}\Delta q$$

where ΔV, Δq, ΔH, and ΔR are estimation errors for the volume, radius, height, and shape parameters of the bowl, respectively, and${\;V}_{0}$, $q_{0}$, $H_{0},$ and $R_{0}$ are the corresponding ground truth values.

Combining Equations (1) and (5), we have $V=\frac{q}{q+2}\pi HR^{2}.$ Thus

(14) $$∆V\approx \frac{q_{0}{R_{0}}^{2}\pi }{q_{0}+2}∆H+\frac{2q_{0}H_{0}R_{0}\pi }{q_{0}+2}∆R+\frac{2H_{0}{R_{0}}^{2}\pi }{{\left(q_{0}+2\right)}^{2}}∆q.$$

Rearrange and simplify:

(15) $$\frac{∆V}{V_{0}}\approx \frac{∆H}{H_{0}}+\frac{2∆R}{R_{0}}+\frac{2q}{{(q}_{0}+2)q_{0}}.$$

Since, for real-world bowls, the choices of parameters q,H, and R are not dependent on each other, we can assume that they are statistically uncorrelated. Hence,

(16) $${\sigma_{V}}^{2}=E\left[{\left(\frac{∆V}{V_{0}}\right)}^{2}\right]-{\left(E\left[\frac{∆V}{V_{0}}\right]\right)}^{2}$$

where $\sigma_{V}$ is the standard deviation of V. Similarly, we can define $\sigma_{q}$, $\sigma_{H}$, and $\sigma_{R}$. Now, let us consider the last term in Equation (16). By the optimization theory, at the neighborhood of the ground truth, the squared error surface function tends to be quadratic [45]. As a result, within this neighborhood, the chances of positive and negative estimation errors tend to be equal. Therefore, we assume that the LM algorithm (Steps 2 and 4 in the second paragraph in Section 2.3) is an unbiased estimator in the neighborhood of the ground truth, i.e., the expected values of these estimates are equal to zero. Using this assumption and substituting Equation (15) for Equation (16) yield

(17) $$\begin{array}{c} {\sigma_{V}}^{2}\approx E\left[{\left(\frac{∆H}{H_{0}}\right)}^{2}\right]+E\left[{\left(\frac{2∆R}{R_{0}}\right)}^{2}\right]+E\left[{\left(\frac{2}{\left(q_{0}+2\right)}\right)}^{2}{\left(\frac{∆q}{q_{0}}\right)}^{2}\right]\\ \approx {\sigma_{H}}^{2}+4{\sigma_{R}}^{2}+\frac{4}{{\left(q_{0}+2\right)}^{2}}{\sigma_{q}}^{2}. \end{array}$$

Equation (17) indicates that the volumetric estimate spreads more widely than each of the H, R, and q estimates (amplification effect). Equation (17) also indicates that $\sigma_{v}$ is more sensitive to $\sigma_{R}$ than $\sigma_{H}$ but for $q_{0}>1$, it is less sensitive to $\sigma_{q}$.

## 3. Experiments

To evaluate our bowl measurement method, we first conducted experiments using both computer-simulated and real-world bowls. Then, the mathematical bowl model was validated using 3D scans of bowls of various shapes. In the computer simulation experiment, all the parameters, including the three parameters for the bowl itself, landmark points on a simulated ruler, and the intrinsic and extrinsic parameters of the camera were all known in advance. They served as the ground truth to facilitate the evaluation of the estimation error resulting from the optimization algorithm. In the second experiment, real-world bowls were evaluated using hand-measured values as the ground truth. We also studied several important practical issues in this experiment, including the choices of a paper ruler, interpolation of the landmark points, and the deviations between the shapes of the modeled and real-world bowls. In the third experiment, we validated the bowl model by comparing the central curve derived from the digitally scanned bowl data with the curve produced by our mathematical model. These studies facilitate practical applications of our method.

### 3.1. Simulated Bowls

Our simulation study included the following steps: (1) constructing a virtual 3D bowl; (2) simulating a virtual paper ruler on the interior surface of the bowl; (3) equally dividing the length of the paper ruler into 20 segments and, for each segment, finding landmark points along the ruler’s two side edges; (4) selecting the extrinsic parameters of the camera (generated randomly in a practical range for each parameter) and projecting the virtual ruler and landmark points onto a 2D image using the ideal pinhole camera model; (5) implementing the optimization algorithm and comparing the results with the ground truth values. The details of these steps are presented below.

The set of bowl parameters $\left\{R,\;H,\;q\right\}$ was generated randomly. After generating each set, a curve $z=f\left(y\right)$ given in Equation (3) was formed. Then, the simulated bowl was obtained by rotating $f\left(y\right)$ around the z-axis, as shown in Figure 1. The total length of ruler L is given by

(18) $$L=2\int_{0}^{R}\sqrt{1+{\left(\frac{df(y)}{dy}\right)}^{2}}dy=2\int_{0}^{R}\sqrt{1+{\left(\frac{y^{q-1}}{R^{q}}Hq\right)}^{2}}dy.$$

**Figure 5:**
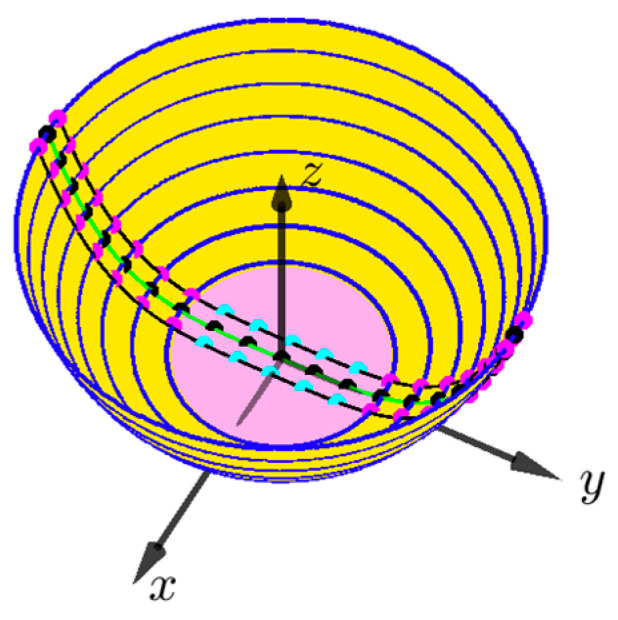
A simulated bowl with a paper ruler in 3D space. Blue lines represent concentric circles perpendicular to the green curve. Magenta dots indicate pairs of landmark points spaced by the ruler’s width (5 mm). Cyan dots in the pink-hatched area near the bottom show points calculated directly by curve length. The z-axis represents the vertical height of the bowl, while the x and y axes define the horizontal plane.

Unfortunately, Equation (18) cannot be written in closed form. We thus evaluated it numerically by accumulating the squared function with small increments of y. Then, we equally divided L into 20 segments and obtained the set of 3D landmark points ($p_{i},$ i=1, 2,⋯,N). The result is shown as the green line in Figure 5. The length of each segment is *l*, as shown in Figure 4c. Since *L* is a function of H,q, and R without a closed form, so are *l* and $p_{i}$. This clarifies the issue mentioned in the first paragraph of Section 2.3. Next, we drew a series of parallel circles on the bowl surface, perpendicular to the green curve (blue lines in Figure 5). Finally, the two points on each circle to both sides of the curve defined a pair of landmark points (each pair of magenta dots in Figure 5). Note that the two landmark points were centered at each division, and the curve length between the pair of points was equal to the ruler width (5 mm in our case). In the simulation process, special care was taken in the area near the bottom of the bowl (pink hatched area), where the concentric circles become very small, and the height difference between neighboring concentric circles is very small, approximately in the same plane. In this area, we calculated the landmark points directly by curve length without using concentric circles (shown as cyan dots in Figure 5).

After obtaining all the 3D coordinates of the landmark points, we used Equations (7) and (8) to obtain the 2D projections of the 3D bowl. In this process, the camera parameters were randomly generated as part of the simulation process. To ensure that the ranges of all the randomly generated parameters were reasonable in practical cases, these ranges, listed in Table 1, were manually specified based on knowledge of real-world bowls.

**Figure 6:**
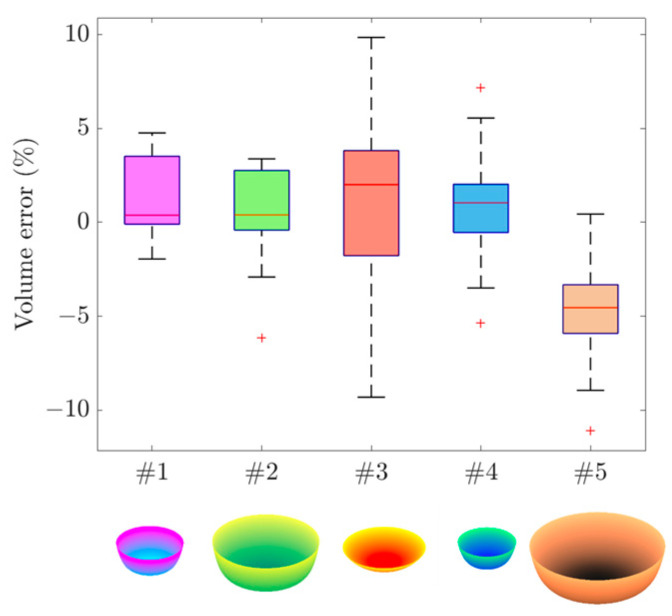
Box plot of simulated bowl volumetric errors. Different colors correspond to different simulated bowls, labeled as #1 to #5. Each box plot is based on 20 images captured from various camera orientations and locations. For each box, the line within the box represents the median of the errors. The bottom and top edges of the box are the first and the third quartiles, respectively, defining the interquartile range (IQR). The extreme regions are defined as 1.5 times the IQR beyond the first and third quartiles, or quartile ± 1.5IQR. Points outside this region are plotted individually as “+”, representing the likely outliers.

Using the methods described above, we simulated five bowls with different shapes and sizes as representatives of real-world bowls. These bowls are graphically shown in the bottom row of Figure 6.

Next, for each bowl, 25 images were generated with random camera extrinsic parameters, including translation distances (X,Y,Z) and Euler angles (ϕ,θ,ψ) within the specified parametric ranges in Table 1. Table 2 shows the true and estimated bowl and camera parameters (rows 2 through 10) from our optimization algorithm. The estimated and true volumes of the bowl are also listed. Note that the estimated values in Table 2 are based on the mean and standard deviations calculated from the 25 independent simulations with random parameters.

It can be observed in Table 2 that our optimization method is highly accurate. Most estimation errors are below 1%, and the maximum error is less than 5%. It can be observed that larger relative errors are usually associated with the estimates of parameter q and volume V. We believe that the q estimates are more difficult than R and H because q is the power of variable y. The large error associated with V is due to an amplification effect, as indicated in Section 2.4. The second-to-last row in Table 2 shows the standard deviation of the estimated volumetric error. The last row in Table 2 provides the calculated volumetric error according to Equation (17). In theory, these errors differ from the true errors by the second and higher-order terms [46].

The five simulated bowls were characterized by different sizes and shapes (bottom row in Figure 6). The box plots in the same figure show the relative volumetric errors of these bowls. It can be observed that the volumetric errors exhibit consistent patterns of distribution across different bowl shapes and sizes. The results also show a few error outliers. When considering all the simulated images (a total of 125), our calculation found that the averaged interquartile range (IQR) of all the volumetric errors was 4.7%, indicating that our algorithm is robust.

Among the five bowls, the box plot for bowl #3 shows a much wider range of IQR. We believe that this was caused by the shape of bowl #3 (the red one in the bottom row), which appears more like a plate than a bowl. For such a “bowl”, the standard deviations of the estimates for radius (R), height (H), and power q tend to be larger, as observed in Table 2.

### 3.2. Real-World Bowls

#### 3.2.1. Paper Ruler Selection

The adhesive paper ruler plays a critical role in our bowl measurement method. We chose yellow as the color of the ruler (Figure 3) because of its sharp contrast with the colors of most bowls, as yellow bowls are relatively rare. Thus, the yellow color facilitates image processing. Adhesive paper rulers on the market typically use either a metric or an imperial scale. Although fine divisions in millimeters produce denser landmark points, our study found that too many landmark points caused excessive complexity in image processing without producing proportional improvement in model accuracy. In addition, at certain camera angles, the fine divisions of the ruler became difficult to identify from the image.

**Figure 7:**
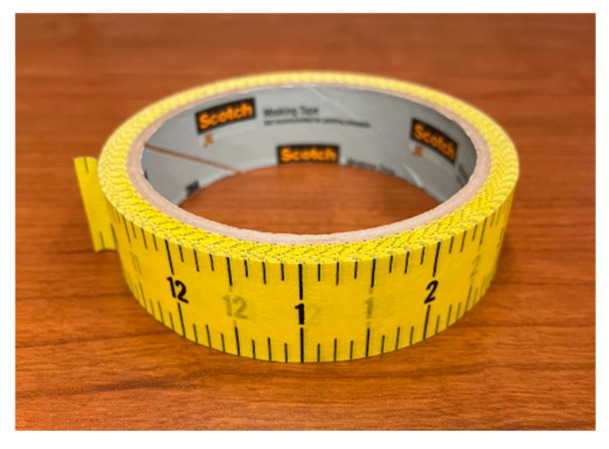
A roll of adhesive paper ruler as a tool for measuring bowls.

As opposed to the metric scale in millimeters, our study shows that 0.125-inch divisions in the imperial system balanced the data processing complexity and measurement accuracy. We also studied the width of the paper ruler. We found that if it was too narrow, the effect of width variation observed in the image was reduced. Conversely, if it was too wide, the curvature of the bowl surface created wrinkles due to uneven local tension in the paper ruler. Considering all these practical factors, we finally chose a yellow color, 0.125-inch divisions, and 0.94-inch width paper ruler. It was made in the form of a low-cost adhesive tape (Figure 7).

#### 3.2.2. Landmark Labeling of Real-World Bowl Image Processing

Identifying the landmark points on the paper ruler is a crucial step before applying our optimization algorithm. Manual identification is feasible and robust but time-consuming. Conversely, automatic identification using an image processing algorithm is fast and labor-free but sensitive to imperfect images, which poses a risk of producing large errors (outliers). Balancing both approaches, we used a semi-automatic method.

**Figure 8:**
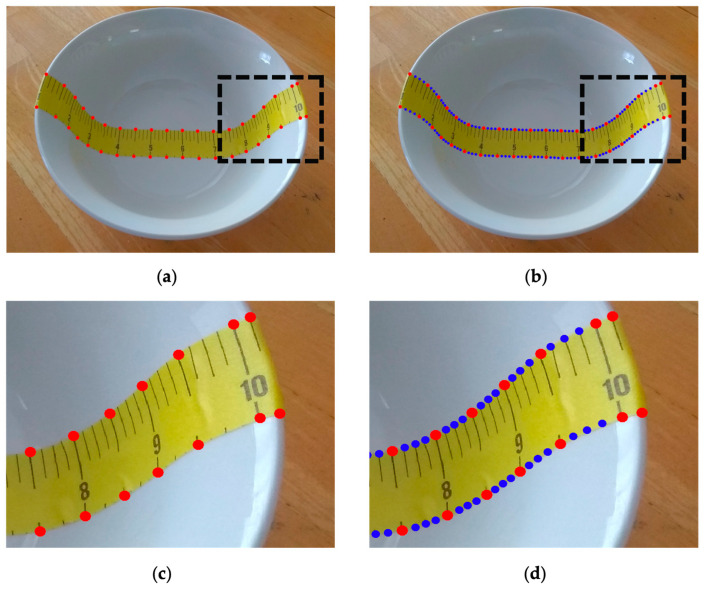
(**a**) Selected landmark points for computation (red points); (**b**) interpolated landmark points (blue points); (**c**) and (**d**) enlarged details within the black boxes in (**a**,**b**).

**Figure 9:**
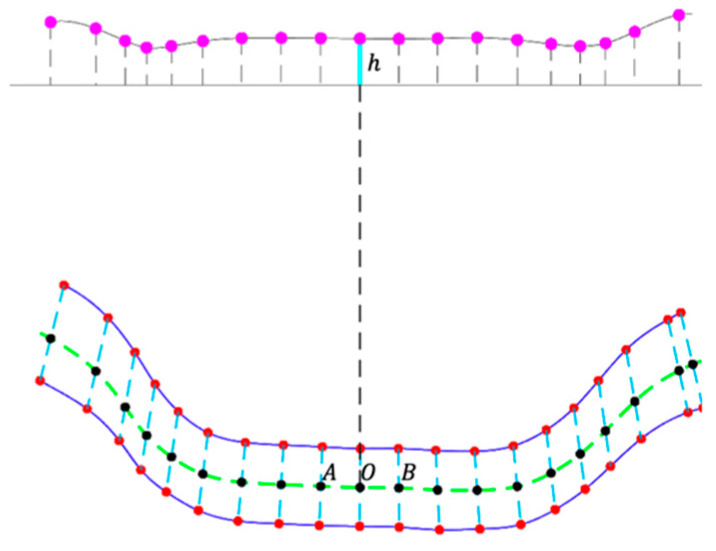
Extracted landmarks on the image. The red points are manually selected, and the black points represent the central landmarks. The height of the top magenta point represents the distance between two neighboring black points.

We first manually labeled the landmark points at intervals of 0.5 inches (red points in Figure 8a). However, the landmarks near the rim of the bowl must be labeled (Figure 8c). To filter out the small, random errors of hand-labeling, we first used a simple averaging filter that produces the midpoints (black points on the green line in Figure 9) along the dashed blue line connecting each pair of red dots (vertical filtering). Similarly, we applied the same averaging filter to the distances between the left and right neighbors (horizontal filtering), i.e., at point O, we computed

(19) $$h=\frac{\bar{OA}+\bar{OB}}{2}$$

where the top bars denote the distances between the corresponding points and h is the filtered distance (plotted in the top row of Figure 9). After these filtering processes, we up-sampled the landmark points by 4, using the existing points (except the bordering ones) to obtain the final result (Figure 8d).

#### 3.2.3. Accuracy of Bowl Parameter Estimation

**Figure 10:**
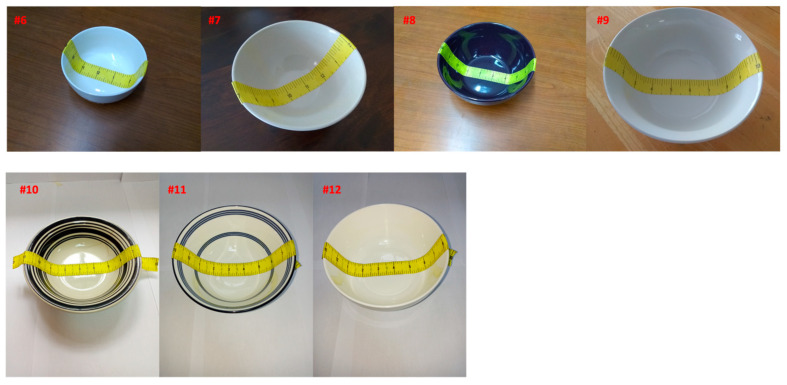
Seven real-world bowls used in the experiments. Bowls #6 to #9 were selected from our laboratory, while bowls #10 to #12 were from East Africa.

We utilized seven real-world bowls (Figure 10). Among them, four were selected from the bowls available in our laboratory (bowls #6 through #9, originally from the authors’ homes). We also selected an “odd-shaped” bowl (#6), which was like a trapezoid in cross-section. The remaining three bowls (bowls #10, #11, and #12) were from our field study conducted in East Africa.

For each bowl, we hand-measured its height and diameter using a ruler and its volume by filling it with water and measuring its weight. To measure q, we calculated the plumpness using Equation (2) based on the measured volume and then used Equation (5) to find the q value. These measurements were used as the ground truth in our experiments.

**Figure 11:**
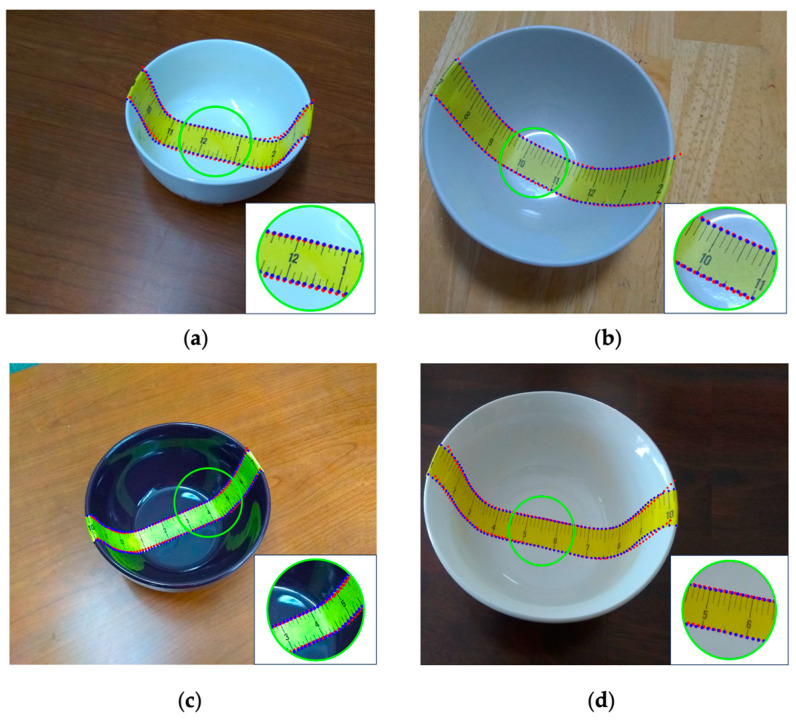
Comparison between the ground truth landmark points (blue) and the estimated landmark points (red) for four real bowls. The comparison results for bowls #6 to #9 are shown in (**a**–**d**).

Next, we used our modeling method to estimate the bowl parameters. The results are shown in Table 3 and Figure 11. It can be observed that the estimated values of the bowl parameters are mostly below 10%, indicating that our model-based estimation approach is generally accurate. It is also noticed from Table 3 that the estimates for bowl #6 tend to be much larger than those for the rest of the bowls. We believe that this reflects the limit of our mathematical model, which cannot represent the shape of this bowl adequately (to be discussed further in the next section).

### 3.3. Validation of the Bowl Model

**Figure 12:**
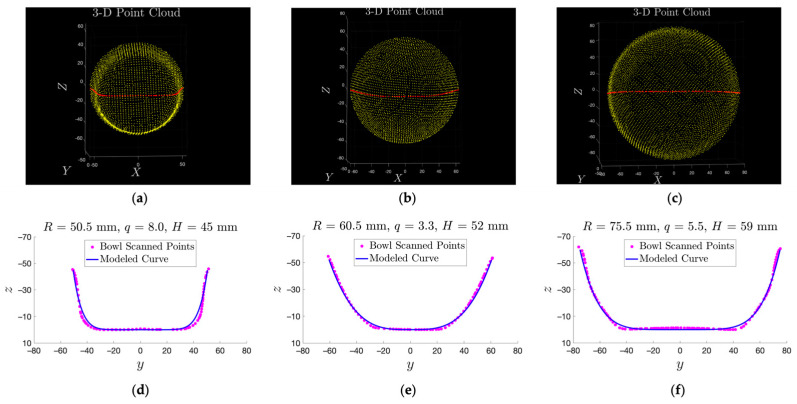
(**a**–**c**) 3D-scanned point clouds from three real bowls, with red points representing the central curves. (**d**–**f**) Comparison between the scanned points along the central curve of the real bowl (magenta) and the modeled (blue) cross-section curves.

To thoroughly evaluate the accuracy of our mathematical model given in Equation (3), we employed a 3D scanner (Model: Revopoint POP 2, Los Angeles, CA, USA) to reconstruct the shapes of three bowls (#6, #7, and #8) and compared these shapes with our model. Figure 12 shows three sets of 3D point clouds resulting from our scans. Next, we sliced each point cloud along the central cross-section (blue curve in Figure 12a–c). The results are shown in Figure 12d–f as red asterisks. Our mathematical model, represented by Equation (3), based on hand-measured parameters, is shown as pink curves. It can be observed that, for bowls #7 and #8, our model and the 3D-constructed point are well-matched. On the other hand, our model fits poorly for bowl #6, which has a trapezoid-like cross-section (Figure 12d). It can be observed that the fitting was worst at the “elbow” areas of the bowl. This indicates that our model is not universally applicable to all bowls. For bowls that cannot be modeled as a power function, the measurement method reported in [29] that does not depend on a mathematical model can be utilized.

Some additional features can be observed in Figure 11d. At the bowls’ top part (near the rim), the bowls usually bend outwards. We believe that this bending improves the bowls’ appearance and makes them more comfortable to hold for self-feeding. Although this small bend is not reflected in our model, our study indicates that the resulting error is not excessive since the bend is usually small.

**Figure 13:**
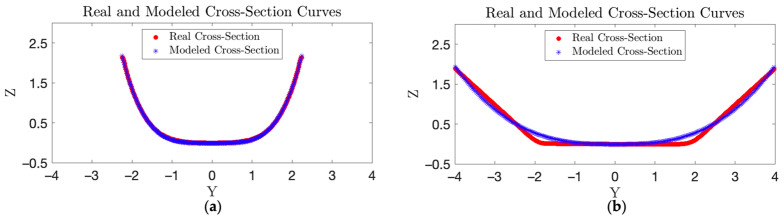
Comparisons between real and modeled bowls in cross-section: (**a**) bowl #20 (name: “IKEA Vardagen bowl,” diameter 4.72”, height 2.36”. (**b**) Bowl #14 (name: “IKEA 365+ deep plate,” diameter 8.66”, height 2.36”.

We further validated our mathematical model using a public set of digital 3D bowl models in the form of point clouds [37]. Most bowls in this dataset were models of commercial bowls sold in IKEA stores. Since the shapes of many bowls were similar, it was unnecessary to model all the bowls. We thus manually selected 10 3D models representing different bowl shapes and sizes. The interior surface of each selected bowl was fitted with our mathematical model. The results (two examples) are shown in Figure 13, and the modeling errors (for all 10 bowls) are listed in Table 4.

While Figure 13a shows an almost perfect fitting between the real and modeled bowls, Figure 13b indicates a relatively inferior fitting since this “bowl”—in fact, a deep plate—cannot be accurately represented by a power function, similar to the case of bowl #6 in Figure 12d. In Table 4, four different types of modeling errors (mean absolute error (MAE), relative MAE, root mean squared error (RMSE), and relative RMSE) are listed, where the two relative errors were both normalized by the bowl height. The data suggest that our bowl modeling method is accurate for most real-world bowls.

## 4. Discussion

In this section, we discuss several important issues, including assumptions, limitations, and applicability of our method to food volume estimation.

Our mathematical bowl model assumes that a bowl’s shape can be modeled as a power function. Our studies have shown that this assumption is acceptable for most real-world bowls. However, as shown in Section 3.3, there are bowls that do not fit the model well. Specifically, bowls with a trapezoidal cross-section do not conform well to this model. Furthermore, bowls with an uneven rim or a rim diameter smaller than the bowl’s maximum diameter pose significant difficulties. Not being able to model all real-world bowls is a limitation of the proposed method.

Additionally, our 3D bowl reconstruction method assumes that the paper ruler adhered to the interior surface of the bowl is fully visible in the image. In case where an occlusion occurs, the image should be re-taken to avoid excessive measurement error.

Although our work is focused on the measurements of bowl geometry, the measurement is critically linked to the estimation of food portion size, a key requirement in dietary assessment. The measured geometry includes bowl diameter, height, and curvature, which directly influences how food occupies space within it. By precisely modeling the bowl’s shape and size, our method provides a reliable utensil reference to determine the volume of food contained within the bowl.

In comparison, the AIM method [47] offers an automated approach to estimating plate and bowl dimensions in dietary assessment through a wearable camera. The integrated ranging sensor is used to provide the distance between the sensor and the eating surface, eliminating the need for a fiducial marker. However, the wearable sensor is required, and the experiment results show that the height estimation is not very reliable due to the large variance. In contrast, our method only requires an image of a bowl with a paper ruler adhered to the interior surface of the bowl. It can estimate not only the bowl’s height and diameter but also its shape (i.e., plumpness). This detailed shape information provides a more accurate reference for estimating the volume of food in the bowl.

## 5. Conclusions

In this work, we have presented a practical method to measure circular dining bowls using an adhesive paper ruler and a smartphone. A robust mathematical model containing three parameters was developed, and an optimization method was implemented to estimate these parameters. The new method allows a smartphone picture to be taken at an arbitrary view angle, which simplifies the bowl measurement process significantly as compared to the previous method. This advancement is particularly important for accurate food volume estimation in dietary assessments, where the size and shape of bowls play a crucial role. To understand the statistical properties of the estimation errors, an error sensitivity analysis was performed. A mathematical expression linking the standard deviations of the bowl volumetric and parametric errors was derived. Finally, several experimental studies were conducted to demonstrate the effectiveness of the proposed bowl measurement method.

## Figures and Tables

**Table 1:** Ranges of randomly generated parameters for both the simulated bowls and camera extrinsic parameters.

*R* (mm)	H (mm)	q	X (mm)	Y (mm)	Z (mm)	θ (°)	φ (°)	ψ (°)
[30, 130]	[9, 130]	[2, 10]	[−400, 400]	[−400, 400]	[180, 500]	[30, 90]	[−60, 60]	[−60, 60]

**Table 2:** Simulation results for five bowls.

	#1	#2	#3	#4	#5
Actual *H* (mm) Estimated *H* (mm) Relative error * (%)	51.0 51.5 ± 0.5 0.9 ± 1.0	71.5 71.6 ± 0.6 0.1 ± 0.8	32.0 32.2 ± 0.5 0.5 ± 1.5	44.0 44.2 ± 0.4 0.4 ± 0.9	78.0 76.6 ± 0.6 −1.7 ± 0.8
Actual *R* (mm) Estimated *R* (mm) Relative error * (%)	64.0 64.3 ± 0.6 0.5 ± 1.0	102.0 102.0 ± 0.8 0.3 ± 0.8	80.0 80.4 ± 1.2 0.5 ± 1.5	55.0 55.2 ± 0.5 0.4 ± 0.9	130.0 128.0 ± 1.0 −1.7 ± 0.8
Actual *q* Estimated *q* Relative error * (%)	6.3 6.3 ± 0.4 −0.6 ± 6.7	9.0 8.9 ± 0.2 −1.1 ± 1.9	3.5 3.5 ± 0.2 0.0 ± 6.6	5.2 5.2 ± 0.3 0.4 ± 6.8	8.5 8.7 ± 0.3 2.5 ± 3.6
Actual volume (cm^3^) Estimated volume (cm^3^) Relative error * (%)	500.0 506.4 ± 10.1 1.3 ± 2.0	1909.0 1921.3 ± 45.6 0.6 ± 2.4	409.0 415.3 ± 17.9 1.4 ± 4.4	302.0 305.3 ± 8.2 1.1 ± 2.7	3352.0 3194.0 ± 79.5 −4.7 ± 2.4
Experimental $\sigma_{V}$ Calculated $\sigma_{V}$	0.020 0.027	0.024 0.018	0.044 0.041	0.027 0.028	0.024 0.019

* Relative error = (estimated value − actual value)/actual value × 100%.

**Table 3:** Simulation results of model parameters for seven real-world bowls.

	Bowl#6	Bowl#7	Bowl#8	Bowl#9	Bowl#10	Bowl#11	Bowl#12
Actual *H* (mm)	45.0	52.0	59	61.0	43.0	65.0	63.0
Estimated *H* (mm)	52.4 ± 2.1	53.6 ± 1.9	63.8 ± 2.7	65.3 ± 2.1	44.8 ± 0.4	64.0 ± 0.0	64.0 ± 0.0
Relative error * (%)	16.4 ± 4.6	3.1 ± 3.6	8.1 ± 4.6	7.0 ± 3.5	4.1 ± 0.8	−1.5 ± 0.0	1.5 ± 0.0
Actual R (mm)	50.5	60.5	75.5	76.0	87.5	76.0	75.5
Estimated R (mm)	52.4 ± 2.1	61.0 ± 1.2	76.0 ± 2.1	71.0 ± 1.2	89.5 ± 0.7	80.0 ± 0.0	80.0 ± 0.0
Relative error * (%)	3.8 ± 4.1	0.8 ± 2.0	0.7 ± 2.8	−6.6 ± 1.6	2.3 ± 0.8	5.3 ± 0.0	5.9 ± 0.0
Actual *q*	8.0	3.3	5.5	4.9	2.3	3.0	5.1
Estimated *q*	8.3 ± 0.4	3.7 ± 0.2	5.9 ± 1.0	7.4 ± 0.6	2.9 ± 0.1	2.9 ± 0.1	4.9 ± 0.1
Relative error * (%)	4.2 ± 5.4	11.5 ± 7.2	8.0 ± 18.7	5.0 ± 13.3	28.2 ± 3.1	−3.3 ± 4.7	−4.9 ± 1.4
Actual volume (cm^3^)	288.0	371.0	773.0	787.0	557.0	705.0	818.0
Estimated volume (cm^3^)	365.6 ± 40.7	405.6 ± 8.7	859.9 ± 34.5	811.5 ± 17.1	671.2 ± 22.4	761.3 ± 15.2	911.1 ± 3.9
Relative error * (%)	26.9 ± 14.1	9.3 ± 2.3	11.2 ± 4.5	3.1 ± 2.2	20.5 ± 4.0	8.0 ± 2.1	11.4 ± 0.5

* Relative error = (estimated value − actual value)/actual value × 100%.

**Table 4:** Modeling errors of 10 dining bowls.

Bowls	#13	#14	#15	#16	#17	#18	#19	#20	#21	#22
MAE (inches)	0.009	0.076	0.017	0.038	0.016	0.029	0.036	0.008	0.026	0.017
Relative MAE (%)	0.397	2.552	0.777	1.240	0.632	1.268	1.541	0.276	1.383	0.777
RMSE (inches)	0.012	0.096	0.029	0.060	0.021	0.044	0.048	0.011	0.038	0.029
Relative RMSE (%)	0.490	2.996	0.968	1.503	0.770	1.546	1.857	0.339	1.685	0.968

## Data Availability

The datasets used and/or analyzed during the current study are available from the corresponding author upon request.
